# A Highly Efficient Biomass Compound Aerosol Suppressant in Purifying Radioactive Cesium Droplet Aerosols

**DOI:** 10.3390/molecules27196480

**Published:** 2022-10-01

**Authors:** Lang Wu, Shuchang Lei, Yixia Wang, Shiyu Yang, Xiaoyan Lin, Haijun Wang

**Affiliations:** 1 School of Materials and Chemistry, Southwest University of Science and Technology, Mianyang 621010, China; 2 Engineering Research Center of Biomass Materials, Ministry of Education, Mianyang 621010, China; 3 The Naval Medical Center, Shanghai 200433, China

**Keywords:** simulated radioactive cesium aerosol, biomass aerosol suppressant, coagulation

## Abstract

Nuclear accidents and decommissioning in the nuclear industry would release a large number of radioactive aerosols which endangers the natural environment and the health of workers. Therefore, there is an urgent need for environment-friendly aerosol suppressants to control and handle environmental pollution problems caused by radioactive aerosols. In this paper, sodium alginate (SA), a type of polyphenol material (TP), and alkyl glycosides (APGs) were selected as the components of the compound aerosol suppressant and the optimal proportion was generated via the method of D-optimal mixture design. Furthermore, the cesium aerosol sedimentation effect of the optimized compound aerosol suppressants was evaluated via sedimentation efficiency, the change in particle concentration cumulative concentration fraction of the cesium aerosol sedimentation process. The results showed that the aerosol sedimentation efficiency was 99.82% which was much higher than nature settlement, 18.6% and water spraying sedimentation, 43.3%. Moreover, after spraying the compound suppressant, it displayed a good effect on settling the cesium aerosol particles with a diameter of less than 1 µm, as the concentration of particles was reduced from 55.49% to 44.53%. Finally, the sedimentation mechanism of the compound aerosol suppressant and cesium aerosol particles, such as the coagulation effect, was analyzed using the particle size distribution.

## 1. Introduction

The radioactive aerosol is a stable colloidal system consisting of solid or liquid particles of radionuclides which are dispersed and suspended in a gas phase environment [1,2]. A great number of radioactive aerosols will be inevitably generated from the nuclear fuel cycle, radioactive waste disposal in the nuclear industry, nuclear weapon tests, discharges from nuclear installations, and nuclear accidents [3,4], such as the Fukushima nuclear accident which leaked ^137^Cs radioactive aerosols [5]. In addition, during the discharge process from nuclear installations such as the decommissioning and decontamination (D&D) of nuclear power plants, it is easy to produce a large amount of radioactive aerosol with high mass concentration to the air and that brings a greater risk of internal exposure to workers, especially when they work in chamber places where radioactive aerosols can be easily accumulated, such as equipment rooms, etc. [6,7].

At present, there have been many purification technologies used to deal with radioactive aerosol pollution in different places, such as physical filtration, electrostatic capture, bubbling washing, pressurized dissolved air flotation and fogging, etc. Regarding the radioactive aerosol pollution in chamber places, the best purification method is the fogging technology which is easy to use and produces little wastage at the same time. More importantly, fog can spread to any corner of a contaminated area, while other purification methods cannot. This method aims to reduce the dose of the radioactive aerosol’s airborne contamination, as well as temporarily fix contamination, via aerosol-suppressant fog which is created from atomizing devices [8,9,10,11]. Additionally, it allows operators to work without entering the pollution area when the purification begins, this will reduce the risk of radioactive exposure which is also one advantages of this method. Therefore, using the fogging technology to purify the radioactive aerosol pollution, especially in the process of D&D of nuclear power plants, has become a trend [10,12]. In order to effectively purify or decontaminate aerosols, the key point is the research on aerosol suppressants. Surfactants, salts, polymers, resin, and bitumen are the six categories of traditional suppressants, and as the raw materials of suppressants they have been widely used in the areas of aerosol suppressants [13]. Although the above aerosol suppressants such as halides and crude oil waste present an effective purify property, the second pollution and difficulty of degradation have become a tricky problem after use. In recent years, a super absorbent resin as a new suppressant material has been developed rapidly due to its good water absorption and viscosity [14], but the cost is too high to be widely applied. Therefore, it is of great significance to explore a type of environmentally friendly and low-cost aerosol-suppressant materials.

In that case, the development of environmentally friendly compound aerosol suppressants derived from the green raw material has become the main trend in the aerosol-suppressants field. This type of aerosol suppressant not only possesses the properties of wetting, bonding, and aggregating with the aerosol particles, but also has a wide variety of raw material sources and low prices [13,15]. Basically, the compound aerosol suppressants used in the fogging technologies are classified into three categories: the mixture of carbohydrates, a compound formula based on glycerol or linseed oil, and other complicated formulas [8,9], among them, the application of the first two methods is more common. However, these types of aerosol suppressants are weak in capturing and fixing the cesium aerosol particles, thus, the aerosol suppression efficiency of these two traditional formulas is not high. As a result, to improve the effect of aerosol sedimentation, there is a need to improve the adhesion and coagulation effect between suppressant particles and cesium aerosol particles, such as enhancing the electrostatic property and chelation, or advancing the wettability of suppressant particles to promote coagulation between particles.

Based on the aforementioned review, a carbohydrate, sodium alginate (SA), a type of small molecular polyphenol material named as TP, and a non-ionic surfactant alkyl glycoside (APG) were selected and blended to prepare the compound aerosol suppressants. Among them, as a hydrophilic biopolymer and a linear anionic polysaccharide polymer of β-(1-4)-D-mannuronic (M-blocks) and α-L-guluronic acid (G-blocks) [16], SA can easily bind with a variety of metal ions via electrostatic, ionic interactions, in covalent-like bonding, redox reactions, and coordination. In addition, SA possesses significant rheological properties such as gelling, viscosifying, and stabilization of dispersions [17]. Moreover, SA is a nontoxic, biocompatible, biodegradable material. Those above properties of SA are beneficial to the purification of cesium aerosol; TP is also a natural, non-toxic, and degradable polymer material with a large number of coordination groups and can strongly complex with most metal ions to form precipitates, which helps to capture and fix aerosol more easily. As a type of green surfactant, APG can reduce the surface tension of aerosol suppressants and enhance the wettability at the same time, ultimately advancing the coagulation and sedimentation of aerosol particles.

**Figure 1:**
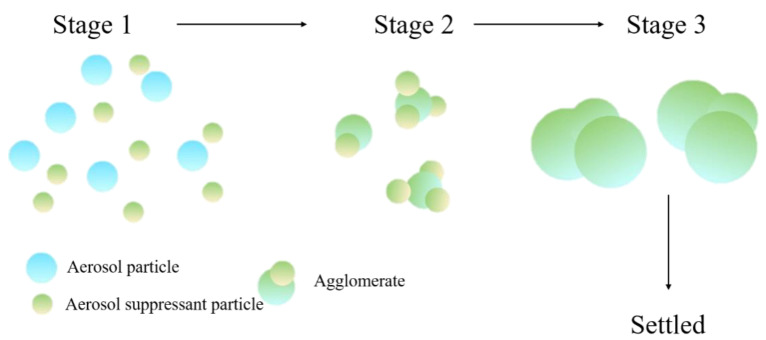
Schematic representation of coagulation and sedimentation of aerosol particles.

The coagulation process of aerosol particles would happen through three simple phases [18], as shown in Figure 1. Firstly, the sprayed aerosol-suppressant particles randomly collide with aerosol particles (stage 1). With the further contact with each other, more and more aerosol particles are captured by aerosol-suppressant particles, and then these two types of particles coagulate with each other and form a larger agglomerate (stage 2). As these agglomerates continue coagulating, the particle size and the weight are increased (stage 3); ultimately, these agglomerates are settled by their own gravity.

Most aerosol measurement methods fall in two categories: the first relies on collection of aerosol particles on a collector, such as filters [19] and some advanced aerosol collectors [20,21], for subsequent laboratory measurements. The second allows in situ, near-real-time measurement of aerosols. Traditionally, collection followed by measurement has been used widely as it can utilize many powerful analytical techniques available in the laboratory [19,22,23]. The sample on the entire aerosol collector can be subjected to gravimetric [23], radioactive [24], chemical [25], or biological [26] analysis, or individual particles on the aerosol collector can be subjected to various forms of spectroscopy, composition [27], microscopy [22], or shape [28] analysis. However, this approach has a disadvantage in that the particles may be modified by the transport and collection processes. Measurements are time averaged, and the measurement result is not immediately available. Real-time techniques, on the other hand, can provide much quicker measurements in situ. For example, the representative APS-3321 aerodynamic particle size spectrometer produced by the TSI Company of the United States can analyze the aerosol particle size in real-time [29], however, they may provide a more limited degree of particle characterization [23,30,31,32,33]. In this study, we used the D-optimal mixture design method to optimize the ratio of each component of compound aerosol suppressants, then particle size distribution and particle concentration of the biomass compound aerosol-suppressant particles, as well as the effects of the biomass compound aerosol suppressant on the particle distribution of simulated cesium droplet aerosols during the sedimentation process were analyzed in situ using the aerodynamic particle size spectrometer (ASP-3321, TSI Instruments, Shoreview, MN 55126, USA). Finally, based on the experimental results, the sedimentation mechanism of simulated radioactive cesium droplet aerosols using a biomass compound aerosol suppressant was analyzed.

## 2. Results and Discussion

### 2.1. Optimization of the Formula of the Biomass Compound Aerosol Suppressant

**Figure 2:**
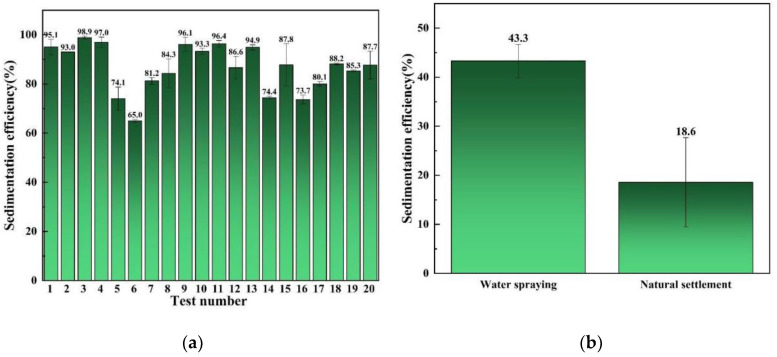
Aerosol settling efficiency: (**a**) the settlement of cesium aerosol by compound aerosol suppressants; (**b**) the settlement of cesium aerosol by water and natural settlement of cesium aerosol.

According to the above review, the biomass materials TP, SA, and APG were selected to prepare the biomass compound aerosol suppressant. Based on the previous experiments, the water, TP, SA, and APG in the compound aerosol suppressant were controlled at 80–89%, 3–10%, 6–7%, and 1–4% respectively. Followed by the experiments listed in Table 1 which were recommended by the D-optimal experimental design, 20 random combinations of water, TP, SA, and APG in the compound aerosol suppressant were used in the aerosol sedimentation tests, and the aerosol sedimentation efficiency of the compound settling suppressant in the 20 random combinations are shown in Figure 2a.

It can be seen in Figure 2 that most of the different combinations of aerosol suppressant presented a favorable aerosol sedimentation efficiency, and the highest aerosol sedimentation efficiency can reach 98.9% which corresponds to the weight fractions of water, TP, SA, and APG of 80%, 9%, 7%, and 4% respectively. This indicated that the combination of TP, SA, and APG exhibited a fine synergistic effect which promoted aerosol sedimentation compared with water spraying and natural settlement. According to the aerosol sedimentation experiment results, the software suggested a special cubic model as the analysis model, which was applied for the analysis of variance (ANOVA) test.

Based on this model, the ANOVA was undertaken to investigate the significance of the model. When the F-value is greater than the p-value and the p-value is lower than 0.05, models are statistically significant at the 95% confidence level [34,35]. The ANOVA analysis of variables tables are shown in Table 2. The p-value of the model was 0.0257 which was less than 0.05, indicating that it was significant and the p-value of the lack of fit, 0.6618, was not significant which was the desired result. In addition, the relationship between the sedimentation efficiency and independent variables was linear with R^2^ = 0.9194, which meant that the model could explain 91.94% of the changes in the range of variables. The coefficient of variation (CV%) was analyzed for the validation of the precision treatment. It helps to identify the level of data spread from the mean. The CV% values ≤ 10% correspond to higher experiment reliability [36], and the CV% value in Table 1 was found to be 6.07%. As a result, it can be concluded that the model was suitable to predict the range of these variables.

Based on the above experiment results, the multiple regression fitting analysis was provided by the software, taking the sedimentation efficiency(y) as the response value and the components of water($x_{1}$), TP($x_{2}$), SA($x_{3}$), and APG($x_{4}$) as the influence factors. The influence of each factor on the response value can be expressed using the following multiple regression Equation (1).

(1) $$\begin{array}{ll} y=-63.59555x_{1} & +2858.42402x_{2}-11489.44370x_{3}-1649.87125x_{4}-34.10349x_{1}x_{2}+132.19628x_{1}x_{3}\\ & +29.09239x_{1}x_{4}-412.48222x_{2}x_{3}+203.43013x_{2}x_{4}+275.12919x_{3}x_{4}+6.48939x_{1}x_{2}x_{3}\\ & -3.82515x_{1}x_{2}x_{4}-3.03316x_{1}x_{3}x_{4}+14.91012x_{2}x_{3}x_{4} \end{array}$$

This equation can be used to compare the relative impacts of the factors by comparing their coefficients. As shown in the regression Equation (1), the absolute value order of the quadratic regression coefficient was $x_{2}x_{3}$, $x_{3}x_{4}$, $x_{2}x_{4}$, $x_{1}x_{3}$, $x_{1}x_{4}$ which indicated that in the compound formula, the combination of TP and SA had the greatest effect on the promotion of the sedimentation of simulated radioactive cesium aerosol, and then followed by the combination of TP and APG, while the combination of water and APG had the weakest effect. According to the analysis results, among all the single components, TP and SA were the most essential ingredients which affected the sedimentation efficiency of the simulated cesium aerosol.

Therefore, based on the previous experiment results, the numerical optimization method was chosen to generate the optimal conditions. We set goals for each variable, as follows: the water was equal to 80%, the TP, SA, and APG were, respectively, in the range of 4–10%, 6–7%, and 1–4%, and the sedimentation efficiency was set to the maximum value. After setting up the parameters mentioned above, the software formed two optimal combinations of the weight fractions of each factor as shown in Table 3. In addition, the predicted value of the sedimentation efficiency of those two combinations was also provided.

Based on the two formulas provided by the optimization function of the software, we prepared two different compound aerosol suppressants which were used for the verification test, and the results in Table 2 showed that the tested value of sedimentation efficiency in the verification test was basically close to the predicted value provided by the optimization function of the software. Moreover, the No.1 test value of aerosol sedimentation efficiency reached 99.82% which was the highest value among all the experiments in this study. Therefore, the optimal weight ratio of different components in the compound settling suppressants was finally selected.

### 2.2. Settlement Characteristics Analysis of the Biomass Compound Aerosol Suppressant

According to the results of the linear regression model analysis, TP and SA played a very important role in the biomass compound settling suppressant. Therefore, the following experiments will focus on characterization the changes between the two raw materials and the biomass compound aerosol suppressant. In addition, the solution concentration of the compound aerosol suppressant, TP and SA, was the same as that used in the aerosol sedimentation experiments.

#### 2.2.1. Aerosol Sedimentation Analysis

**Figure 3:**
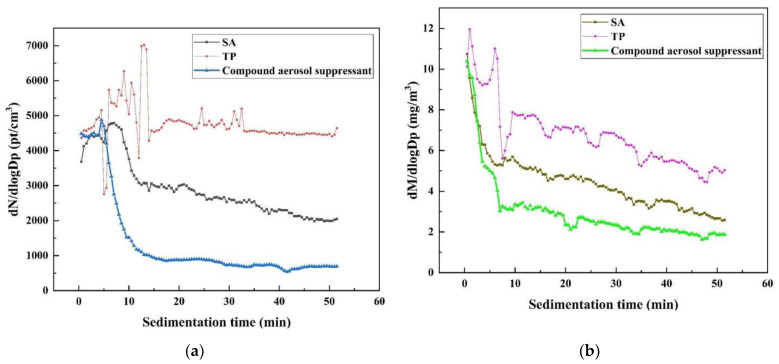
Change in aerosol particle concentration over time: (**a**) change in aerosol particle number concentration over time after spraying the aerosol suppressant; (**b**) change in aerosol particle mass concentration over time after spraying the aerosol suppressant.

Figure 3a shows the log-normal distribution function of the aerosol particle number concentration of cesium aerosol sedimentation, which can describe the change in concentration regardless of the particle size channel resolution, after spraying three different materials. It is obvious that among these three, the compound aerosol suppressant presented the best aerosol purify effect when after sedimentation for 10 min the concentration of aerosol particles showed a sharp decreased from about 4500 to 1000 pt/cm^3^, while SA showed a small suppression effect on the aerosol particles. Moreover, TP almost had no effect on decreasing the concentration of aerosol particles.

After spraying three different materials, the changes in the cesium aerosol particle mass concentration over time shows almost the same trend or pattern as the concentration changes over time as shown in Figure 3b, the log-normal distribution function of the aerosol particle mass concentration of cesium aerosol sedimentation. Again, the purification effect of the composite aerosol suppressant on the cesium aerosol is best among the three suppressants, and the the curve of the aerosol particle mass concentration over time is located at the bottom. After sedimentation for 10 min, the aerosol particle mass concentration shows a sharp downward trend from about 10.5 mg/m ^3^ down to 2 mg/m ^3^. The purification effect of SA on cesium aerosol was the second, and the mass concentration of aerosol particles decreased from about 10.5 to 3.5 mg/m^3^, while the purification effect of TP on cesium aerosol was the worst: the curve of the aerosol particle mass concentration over time is located at the top and the mass concentration of aerosol particles decreased from about 10.5 to 5.5 mg/m^3^. The results of the change in aerosol particle number concentration and mass concentration over sedimentation time all prove that the compound aerosol suppressant has the most outstanding effect on the purification of cesium aerosol, and the compound aerosol suppressant can effectively capture cesium aerosol particles and thus reduce the concentration of cesium aerosol.

#### 2.2.2. Surface Tension Analysis

**Figure 4:**
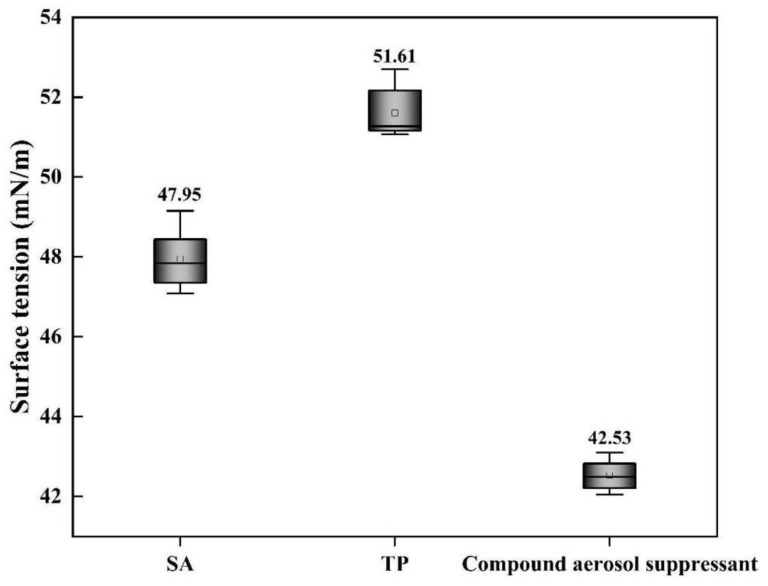
Surface tension of different aerosol-suppressant solutions.

As shown in Figure 4, the surface tensions of SA and TP solutions were 47.95 and 51.61 mN/m, respectively, while the surface tension of the compound component settling suppressant solution was the lowest, only 42.53 mN/m, which was owing to a certain dosage of surfactant APG added into the compound settling suppressant that decreased the solution surface tension.

With the decrease in the aerosol-suppressant solutions’ surface tension, the wettability of the compound aerosol suppressant was improved so that the aerosol-suppressant fog could easily interact with the particles of cesium droplet aerosol, then coagulated and settled eventually and that is one of the reasons why the compound aerosol suppressant showed an extraordinary aerosol-suppression effect.

#### 2.2.3. Viscosity Analysis

**Figure 5:**
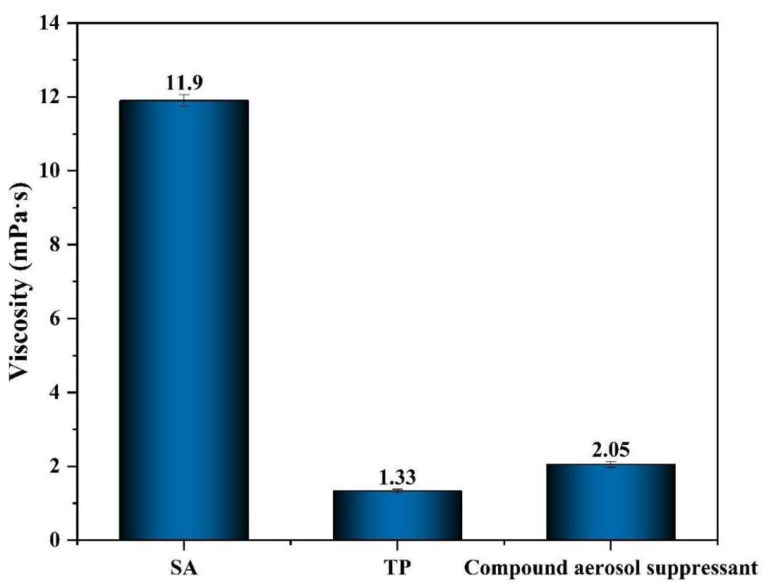
Viscosity of different aerosol-suppressant solutions.

As shown in Figure 5, the viscosity of SA was 11.90 mPa·s, which was the highest among these three different materials, due to its rheological properties of viscosifying. The viscosity of the compound settling suppressant and TP were only 2.05 and 1.33 mPa·s. It is obvious that the viscosity of the compound aerosol suppressant decreased sharply, compared with SA, while compared with TP the value of viscosity increased slightly due to the addition of SA and that improved the adhesion effect between the compound aerosol-suppressant particles and aerosol particles, which promoted aerosol sedimentation. That explained the reason why the aerosol suppression effect of SA was better than TP.

#### 2.2.4. Particle Size Distribution Analysis

**Figure 6:**
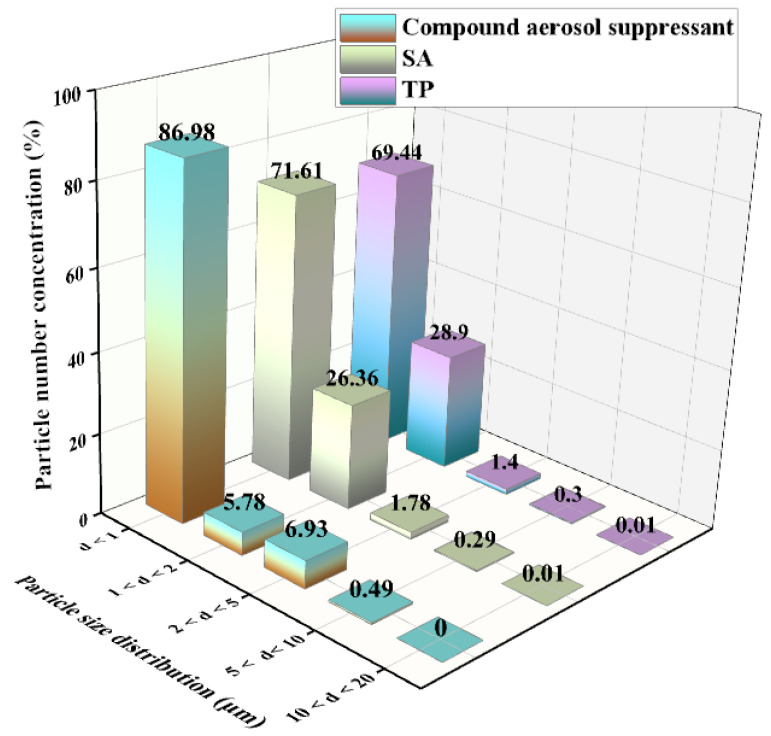
Particle size distribution of different aerosol suppressants after spraying into the sedimentation chamber.

We tested the aerosol suppressant fog particle size distribution. As shown in Figure 6, the test result displayed the particle number concentration fraction of each size. It indicated that basically all the three types of fog particle size were below 5 μm, and most of the particles were below 1 μm. Compared with SA and TP, when the fog particle diameter was less than 1, the particle number concentration of the compound aerosol suppressant was the highest which reached 86.98%, while SA and TP only reached 71.61% and 69.44%. This was due to the addition of the APG surfactant which plays an important role in liquid drop breakup via atomization [37]; research has shown that the surfactant can avoid the coalescence of atomization particles at the spray moment, and that promotes a better atomization effect. Based on the above experimental results, it indicated that compared with SA and TP, the compound aerosol-suppressant fog mainly consisted of smaller particles in which most of the particles were below the diameter of 1 μm, and that effectively improved the ability to capture cesium aerosol particles and explained the high aerosol sedimentation efficiency of the compound aerosol suppressant.

### 2.3. Sedimentation Mechanism Analysis of the Simulated Cesium Aerosol

Based on the previous experimental results, which indicated that the viscosity and surface tension of the aerosol suppressant had a certain effect on aerosol sedimentation, we analyzed the aerosol particle distribution during aerosol sedimentation experiments and collected the aerosol solution samples to study the aerosol sedimentation mechanism of the aerosol suppressant.

#### 2.3.1. Mechanism of Aerosol Particles Captured by Aerosol-Suppressant Droplets

**Figure 7:**
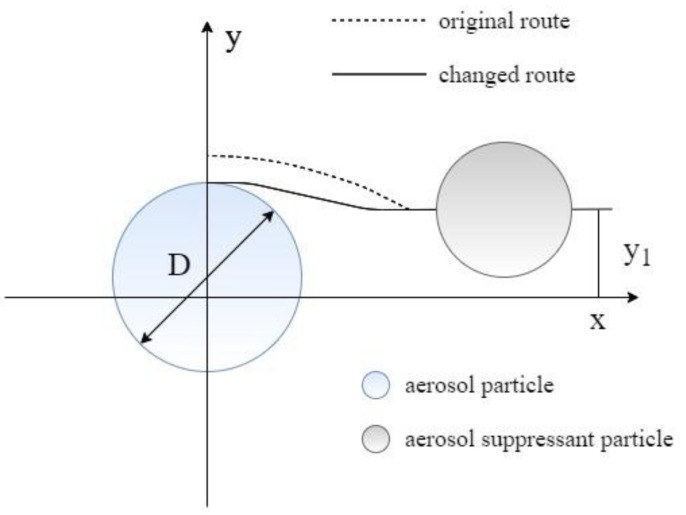
Aerosol particle captured by an aerosol-suppressant particle.

As shown in Figure 7, according to the aerodynamics capture principle of aerosol particles [38], the aerosol-suppressant fog particles have an effect on aerosol particles via diffusion, collision, and coagulation, which changes the original motion trajectory of the aerosol particles and are then captured by the fog particles. The overall efficiency of a single aerosol-suppressant fog particle to capture aerosol particles (E_R1_) can be calculated using the area of a circle with a radius of y_1_ to the projected area of the aerosol-suppressant particles, as shown in Equation (2)

(2) $$E_{R1}=\frac{\sigma \pi y_{1}^{2}}{\frac{1}{4}\pi D^{2}}=\frac{4y_{1}^{2}\sigma }{D^{2}}$$

where D is the diameter of aerosol-suppressant fog particle, m; σ is the coefficient of the aerosol-suppressant fog particle hitting the captured aerosol particles.

**Figure 8:**
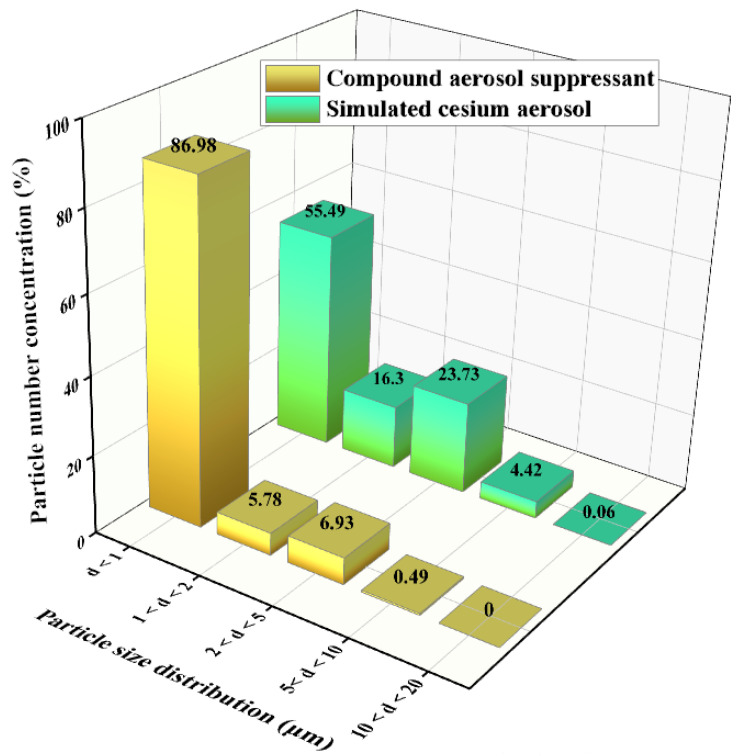
Particle size distribution of the simulated cesium aerosol droplets and compound-suppressant droplets in the sedimentation chamber.

As shown in equation (2), the E_R1_ can be increased through decreasing the aerosol-suppressant particle size, therefore, the aerosol-suppressant fog particles with a smaller size are more conducive to capture of the aerosol particles. Thus, we separately tested the particle size distribution of aerosol-suppressant fog and simulated cesium droplet aerosol, and the test result of the particulate number concentration fraction is shown in Figure 8. The results showed that about 86.98% of the compound aerosol-suppressant fog particles were below the size of 1 μm. While the particle distribution of cesium particles was slightly wider than that, with the majority distributed in the range of less than 5 μm, and only 55.49% were under the size of 1 μm. The contents of particles with a particle size located in the ranges of 1–2 and 2–5 μm were both higher than that of the compound aerosol-suppressant fog particles. It indicated that the compound aerosol suppressant can capture cesium droplet aerosol particles, based on the size and number of fog particles created by the compound aerosol suppressant.

#### 2.3.2. Coagulation and Sedimentation Process

**Figure 9:**
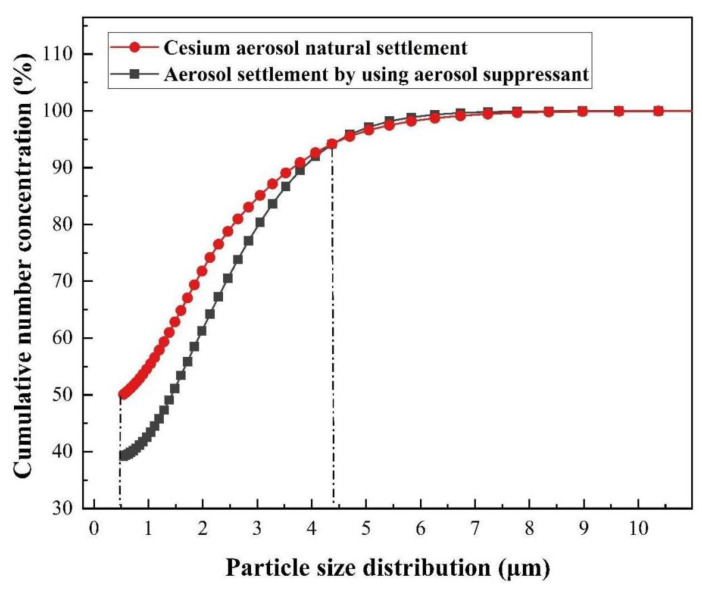
Agglomeration effect of the compound aerosol suppressant.

The theory of the coagulation process reveals that with the process of aerosol sedimentation, the diameters of agglomerates keep increasing. Thus, in order to study the process of increasing agglomerates, we monitored the particle size distribution of the whole process of the cesium aerosol sedimentation, and the results of the cumulative number concentration fraction is shown in Figure 9. It can be seen that when the particle size was between 0.5 and 4.3 μm, compared with cesium aerosol natural settlement, after using the compound aerosol suppressant, the curve obviously moved down and when the cumulative fraction of the number concentration was 50% the particle size apparently increased from 0.5 to 1.5 μm. It indicated that droplet particles interacted wit and adhered to each other, and then formed agglomerates with larger particle diameters. In order to more intuitively see the coagulation effect of the compound aerosol-suppressant on cesium aerosol particles, the content in different particle ranges was further analyzed.

**Figure 10:**
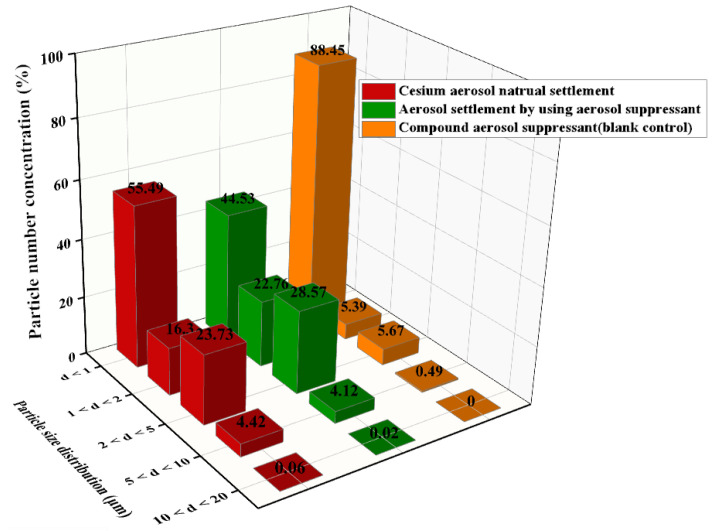
Particle size distribution of the simulated cesium droplet aerosol in natural settlement, the settlement by the suppressant, and the particle size distribution of the compound aerosol suppressant (blank contrast) after spraying into the sedimentation chamber.

As shown in Figure 10, compared with the simulated cesium droplet aerosol in natural settlement, after spaying the compound aerosol suppressant, the concentration fraction of particles with a diameter of less than 1 μm was reduced from 56.61% to 44.53%, which revealed that the compound aerosol suppressant was beneficial for coagulating and settling fine cesium aerosol particles. In addition, compared with the compound aerosol suppressant (blank contrast) after spraying into the sedimentation chamber, the concentration fraction of particulates, in which the particle size was between 1 and 5 μm, significantly increased in the cesium aerosol sedimentation experiment of spraying the compound aerosol suppressant. On the contrast, the particulate number concentration fraction decreased from 88.45% to 44.53% when the diameter was below 1 μm. It indicated that a large number of tiny compound aerosol suppressant particles coagulated with the cesium aerosol particles and then formed into more agglomerates with larger diameters. Therefore, the number concentration fraction of particles with a smaller diameter decreased while other particles with a larger diameter increased, which proved the coagulation process of the compound aerosol suppressant and cesium aerosol particles.

#### 2.3.3. Electric Double Layer Compression and Electric Neutralization

**Figure 11:**
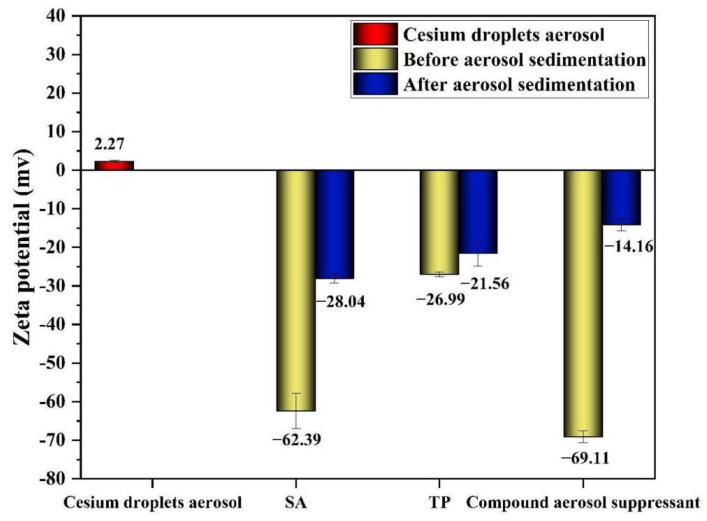
Zeta potential of cesium droplets before and after aerosol deposition by spraying suppressants.

According to the previous experimental results, the compound aerosol suppressant particles showed a coagulation effect on cesium aerosol particles via randomly colliding with each other. Thus, in order to determine whether the compound aerosol suppressant had another effect on cesium aerosol to promote coagulation, we collected the solution samples of cesium aerosol after sedimentation, to test the zeta potential. In addition, SA, TP, and the compound aerosol suppressant were considered as the contrasts and the test results are shown in Figure 11. As it was shown in Figure 11, the zeta potentials of these three aerosol suppressants decreased, compared with the solution samples after aerosol sedimentation. Among the change values of the zeta potential, the change in the compound aerosol suppressant was the most obvious which decreased from 69.11 to 14.16 and was followed by SA which decreased from 62.39 to 28.04. Due to the addition of SA, the compound aerosol suppressant ionized some sodium ions and generated polymer chain segments with anions when dissociated in the water, so that the compound aerosol suppressant fog particles could capture cesium aerosol particles which had a positive charge via the effect of electric neutralization. Furthermore, some positive charge ions squeezed into the stern layer of the colloidal particles causing the compression of the electric double layer which also reduced the zeta potential [39,40]. Moreover, it was reported that the zeta potential, either positive or negative, is generally required to ensure stability, and when the zeta potential decreased, the particles tended to destabilize and agglomerate together, and then settled at last [41]. Thus, based on the above experimental results, the joint action of electric neutralization of the particles and the electric double layer compression promoted the coagulation of the compound aerosol suppressant particles and cesium aerosol particles.

## 3. Materials and Methods

### 3.1. Materials

Sodium alginate (SA) was purchased from Chengdu Kelong Chemical Co., Ltd. (Chengdu, Sichuan, China). Cesium chloride was purchased from Shanghai Macklin Biochemical Co., Ltd. (Shanghai, China). Alkyl glycoside (APG) was purchased from Shanghai Fine Chemical Co., Ltd. (Shanghai, China). Polyphenol material (TP) was purchased from Shanghai Oriental Bojin Biotechnology Co., Ltd. (Shanghai, China).

### 3.2. Preparation of Biomass Compound Aerosol Suppressant and Optimal Design

#### 3.2.1. Preparation of Compound Aerosol Suppressant

The detailed preparation steps were as follows: Firstly, the deionized water, TP, SA, and surfactant with weight ratios according to Table 1 were added into the beaker. Secondly, they were stirred and heated to 35–40 °C for 5–10 min, until the mixture was completely dissolved and well mixed, then the biomass compound aerosol suppressant was prepared. Considering that TP, SA, and APG were all biomass materials, the preservation of sodium benzoate with the dosage of 0.02% was added to the compound aerosol suppressant according to the GB2760-1996 Food Additive Hygienic Standard (1).

#### 3.2.2. Optimal Design

A multivariate optimization procedure was applied using the D-optimal mixture design method which is a prevalent iterative algorithm that minimizes the covariance matrix of the parameter estimates for a certain proposed model [42] and was used to screen out the optimal ratio of each independent variable. The sedimentation efficiency was adopted as the dependent variable. The constraint conditions of the four independent variables were as follows: the deionized water, TP, SA, and surfactant with the weight ratios of 80–89%, 3–10%, 6–7%, and 1–4%, respectively. Table 3 lists 20 randomized experiments and the effects of interactions between variables on each response were determined using this software. All statistical analyses were carried out with the Design Expert software (Version 8.0.6).

### 3.3. Characterization of the Biomass Compound Aerosol Suppressant

The surface tension of the compound aerosol suppressant solution was measured using a surface tension tester (K100, Kruess Scientific Instrument, Hamburg, Germany). Before and after aerosol sedimentation, the zeta potential changes of the compound aerosol suppressant solution were tested with the zeta potential analyzer (Brookhaven Instruments, New York, NY, USA). The viscosity of the compound aerosol suppressant solution was tested using the digital viscometer (NDJ-8S, Jingtian electronic instrument, Shanghai, China). In order to describe the coagulation process of the simulated cesium aerosol particles more precisely, the log-normal distribution function of the aerosol particle size distribution during the sedimentation process was provided using the aerodynamic particle size spectrometer (ASP-3321, TSI Instruments, USA).

### 3.4. Sedimentation Experiment

#### 3.4.1. Simulation of the Cesium Droplet Aerosol

**Figure 12:**
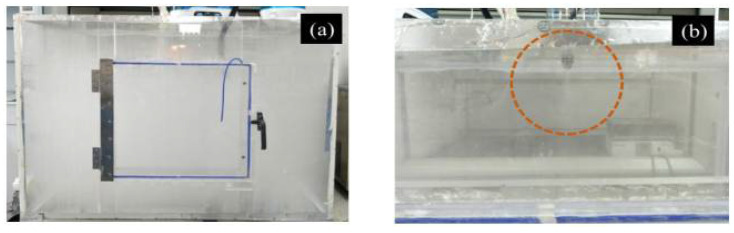
Simulated aerosol device: (**a**) simulation box filled with aerosol; (**b**) spraying effect of the atomizing nozzle.

In order to simulate the radioactive cesium aerosol, firstly, as the radioactive cesium source, cesium chloride solution was added into the ultrasonic atomizer, then the simulated radioactive cesium aerosol was generated and diffused into the simulation box, with a length, width, and height of 100, 80, and 65 cm, respectively, via a pipeline. Finally, the droplet aerosol was formed, as shown in Figure 12a.

##### Sampler Device

**Figure 13:**
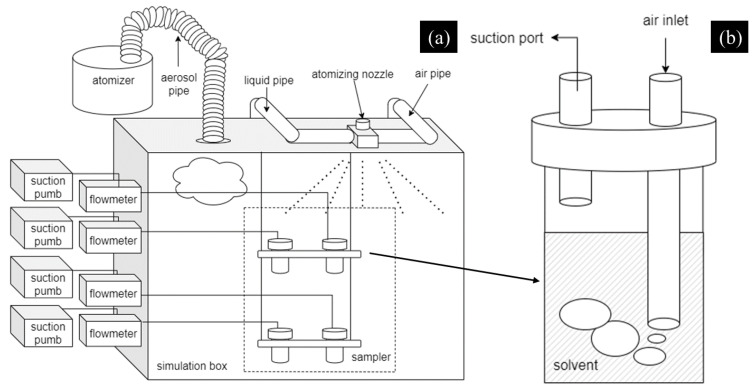
Schematic diagrams of the experimental system: (**a**) simulated aerosol sedimentation system; (**b**) sampler device.

The sampling point was set in the middle of the box, as shown in Figure 13a. Based on the principle of the atmospheric sampler, we designed a type of gas sampler, as shown in Figure 13b. Two different length tubes were inserted into a sampler with a cover. The simulated aerosol was drawn into the sampler through the long tube using a suction pump (2XZ-4, Linhai Tan vacuum equipment, Zhejiang, China) and then dissolved in deionized water to form a solution of cesium ions. Then, the concentration of the cesium ion in the solution samples was measured with the atomic absorption spectrophotometer (Z-2300, Hitachi, Tokyo, Japan).

#### 3.4.2. Aerosol Sedimentation Test

The sedimentation test of the simulated cesium aerosol was carried out in the simulation box, as shown in Figure 13a. After the simulation box was filled with droplet aerosol, the prepared aerosol-suppressant solutions with the volume of 100 mL were sprayed into the simulation box (100 × 80 × 65 cm) via the atomizing nozzle device (commercially available), as shown in Figure 12b and Figure 13a. Then, the gas sampler collected solution samples at the beginning and end of the aerosol sedimentation tests which were recorded as the initial concentration, *C*_0_ and the final concentration, *C*_t_. In each aerosol sedimentation test, the duration of each sampling time was 2 min, the flow rate of the suction pump was 2 L/min, and the atomization pressure of atomizing nozzle was 0.05 Mpa. Moreover, we used the aerodynamic particle size spectrometer to monitor the whole process of the aerosol sedimentation to present the change in the concentration of aerosol particles.

#### 3.4.3. Aerosol Sedimentation Efficiency

The sedimentation efficiency of the simulated cesium aerosol is defined as the change value of the cesium ion concentration (*C*_0_ − *C*_t_) accounting for the fraction of the initial cesium ion concentration (*C*_0_), which was calculated based on the following equation:

(3) $$Sedimentation\;efficiency\left(\%\right)=\left[\left(C_{0}-C_{t}\right)/C_{0}\right]$$

where *C*_0_ and *C*_t_ are defined as the initial and the final concentrations of the simulated cesium aerosol solution samples, respectively.

## 4. Conclusions

In this study, we mainly studied the sedimentation of the simulated radioactive aerosol at room temperature in the confined space of 0.5m^3^ and the effect of the aerosol suppressant on it in this confined space. The simulated cesium aerosols were collected using a suction pump before and after the use of aerosol suppressants, then dissolved in deionized water, the concentration of cesium ions was measured with an atomic absorption spectrophotometer, and the sedimentation efficiency of the aerosol was calculated from the concentration of cesium ions. Based on the fogging technology, we designed a sedimentation system and prepared a low-cost, environmentally friendly biomass compound aerosol suppressant with a high sedimentation efficiency, which consisted of SA, TP, and APG and the optimal formula was generated using the method of the D-optimal mixture design. In addition, the emitted simulated cesium droplet aerosol, the sedimentation properties of the biomass compound aerosol suppressant, the particle size distribution, the particulate number concentration, and mass concentration fraction during the aerosol sedimentation process were further studied using the TSI APS-3321 aerodynamic particle size spectrometer. The detailed conclusions are as follows:

(1) According to the particulate number concentration fraction, the simulated radioactive cesium aerosol particles emitted by ultrasonic atomizers at room temperature are mainly distributed in 1–5 μm, of which 55.49% are less than 1 μm, 16.30% are 1–2 μm, and 23.73% are 2–5 μm. Aiming at the simulated cesium aerosol with this particle size distribution, different materials were used to spray it. The results show that compared with the single components including SA and TP, the compound aerosol suppressant showed good aerosol suppression properties: surface tension of 42.53 mN/m, viscosity of 2.05 mPa·s, and the aerosol sedimentation efficiency reached 99.82% during 30 min at room temperature, meaning it had a significant effect on purifying the cesium aerosol.

(2) According to the particulate number concentration fraction, the compound aerosol suppressant fog mainly consisted of tiny particles in which 80% of particles were below the diameter of 1 μm, and fog particles with a smaller size were more effective at capturing cesium aerosol particles.

(3) The cumulative number concentration analysis of cesium showed, after spraying the compound aerosol suppressant, that the particle size increased from 0.5 to 1.5 μm, which showed an obvious coagulation process.

(4) Based on the tests results, the zeta potential of the compound aerosol suppressant decreased from 69.11 to 14.16, which indicated that the joint action of electric neutralization of the particles and electric double layer compression also promoted the coagulation of the compound aerosol suppressant particles and cesium aerosol particles.

There are many other factors in the environment of the confined space that play a role in the settling of aerosol particles, including temperature, wind speed, humidity, etc. The temperature is the major factor for the sedimentation efficiency of cesium aerosol in this study. The sedimentation efficiency of cesium aerosol was obtained at room temperature in this study. Other factors will be refined in future research.

## Figures and Tables

**Table 1:** ANOVA analysis of variables tables.

Source	Sum of Squares	df	Mean Square	F Value	*p*-Value (prob > F)	
Model	1940.22	13	149.25	5.27	0.0257	Significant
Linear mixture	236.75	3	78.92	2.78	0.1322	
Lack of fit	7.04	1	7.04	0.22	0.6618	Not significant
R^2^ = 0.9194	R^2^_adj_= 0.7448	C.V.% = 6.07				

**Table 2:** The predicted and tested results of the best combination of different ingredients.

Number	ω%				Aerosol Sedimentation Efficiency %	
	Water	TP	SA	APG	Predicted Value	Tested Value
1	80.000	TP_1_	6.125	APG_1_	99.975	99.8220
2	80.000	TP_2_	6.905	APG_2_	98.674	98.3448

**Table 3:** D-optimal experimental design for the compound settling suppressant optimization.

Run	Independent Variables			
	ω%			
	A:Water	B: TP	C:SA	D:APG
1	89.000	3.000	6.000	2.000
2	83.250	6.250	6.500	4.000
3	80.000	9.000	7.000	4.000
4	86.750	4.750	6.250	2.250
5	83.400	7.000	7.000	2.600
6	82.250	8.250	6.250	3.250
7	89.000	3.000	7.000	1.000
8	85.050	6.250	6.250	2.450
9	80.000	10.000	6.000	4.000
10	80.000	10.000	6.000	4.000
11	82.000	10.000	7.000	1.000
12	89.000	3.000	7.000	1.000
13	82.000	10.000	7.000	1.000
14	86.000	3.000	7.000	4.000
15	81.250	10.000	6.500	2.250
16	87.500	3.000	7.000	2.500
17	85.250	4.750	6.750	3.250
18	86.000	7.000	6.000	1.000
19	86.000	7.000	6.000	1.000
20	80.000	9.000	7.000	4.000

## Data Availability

Not available.

## Sample Availability

Samples of the compounds are not available from the authors.
